# Primary Oral Myiasis: A Case Report

**DOI:** 10.1155/2012/734234

**Published:** 2012-10-22

**Authors:** Nitin Bhola, Anendd Jadhav, Rajiv Borle, Nitin Adwani, Gaurav Khemka, Pretti Jadhav

**Affiliations:** ^1^Department of Oral and Maxillofacial Surgery, Sharad Pawar Dental College and Hospital, Maharastra, Wardha 442101, India; ^2^Department of Periodontology and Implantology, Sharad Pawar Dental College and Hospital, Maharastra, Wardha 442101, India

## Abstract

Myiasis commonly refers to invasion of live human or animal tissue by fly larvae of the Diptera order where they complete their cycle totally or in part, feeding on living or dead tissue, as well as on body fluids. Infestation of tissues of vertebrate species is pandemic but more frequently found in tropical and subtropical countries where poor hygiene, poor housing infrastructure, warm humid climate, and proximity with domestic animals prevail. Its diagnosis is made basically by the presence of larvae. The present paper reports a case of gingival myiasis involving 14–16 larvae in a 12-year-old boy.

## 1. Introduction 

Human race has been plagued with parasitic infection since the time immortal. One of the most daunting infestations amongst them through common flies is Myiasis. Myiasis, a term, introduced by Hope (1940) [1], refers to invasion of tissues, organs, and certain body cavities of live human vertebrate species by the dipteran eggs or larvae which commonly manifests as furunculoid lesions. Zumpt [2] defined Myiasis as an infestation of live human and vertebrate animals by dipterous larva, which at least for certain period of its lifecycle feeds on host's dead or living tissue, liquid body secretions, and fluids or ingested food.

It is pandemic; prevalence is specifically related to latitude, lifecycle of various species of flies, and third world countries. Higher incidence is reported in tropical, subtropical regions of Africa, America, and South East Asia, where warm, humid climate prevail almost throughout the year; commonly seen amongst people where sanitation, personal hygiene is often ignored and close association with domestic pets exists. Infestations are common phenomenon in skin, nose, eye, lung, ear, anus, and vagina but oral manifestation is exceptional [3]. Incidence of oral myiasis as compared to that of cutaneous myiasis is less as the oral tissues per se are not permanently exposed to the external environment [4].

Oral myiasis is more commonly ascribed to predisposing anatomical, medical conditions where oral cavity is being exposed to external environment for a prolonged time, mouth breathing, anterior open bite, incompetent lips, cerebral palsy [5], following tooth extractions [6], neglected mandibular fracture [7], patients undergoing mechanical ventilation [8], and certain local pathological conditions such as cancrum oris [4], and oral malignancies [9].

We present a case of oral myiasis in the maxillary anterior region in a patient with neurological deficit.

## 2. Case Report

**Figure 1:**
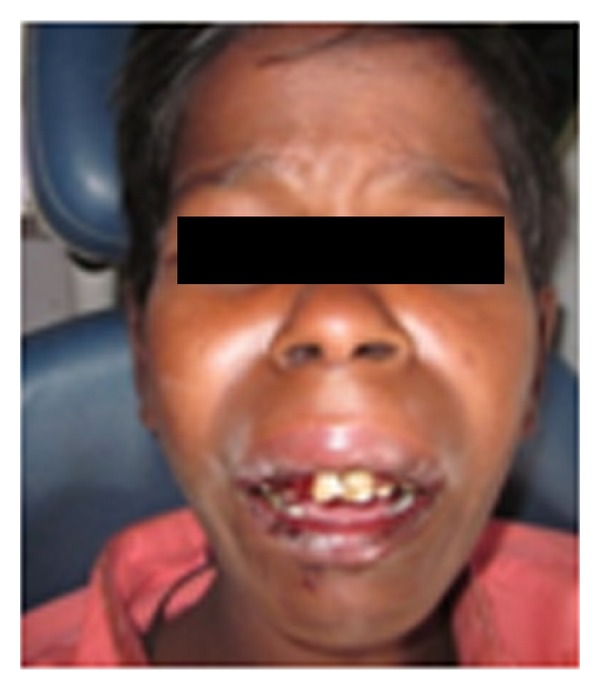
Swelling of the upper lip.

A young 12-year-old male child with neurological deficit reported with his father with chief complains of swelling of upper lip and jaw, discomfort with upper front teeth region since 4-5 days. On examination the patient was moderately built with waddling gait. Extra oral examination revealed incompetent lips, a solitary, large, diffuse, swelling of size approximately 4 × 3 in size associated with upper lip with overlying skin tense, and shiny (Figure 1). The swelling was tender to palpation, soft, and edematous. Intraoral examination revealed mutilated labial gingiva in the region of the maxillary incisors with multiple fenestrations.

**Figure 2:**
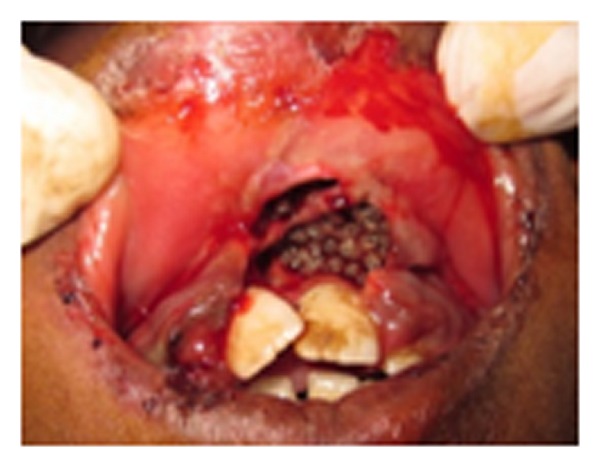
Multiple worm-like motile organisms within the ulcerated area.

The anterior labial gingiva showed a poorly defined swelling measuring 3 × 1 cm with detachment and exposure of underlying bone. Deep burrowing, with multiple cavitations, was seen. Multiple larvae were noted crawling within the gingival lesion (Figure 2). The surrounding mucosa was inflamed and tender to palpation but bleeding and discharge were not evident. Based on clinical findings and presence of maggots, a provisional diagnosis of oral myiasis was made.

**Figure 3:**
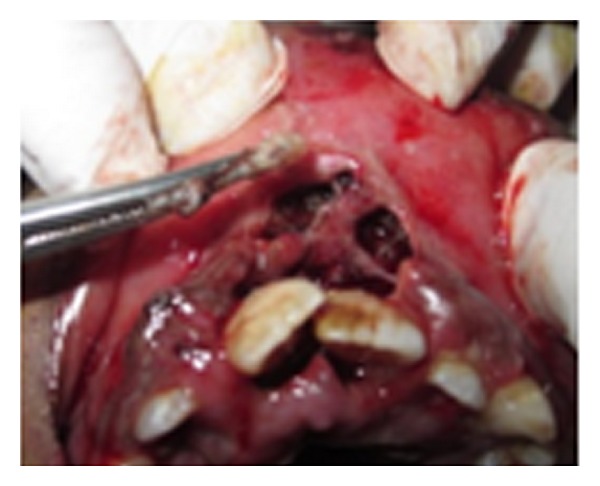
Removal of larvae using haemostat.

**Figure 4:**
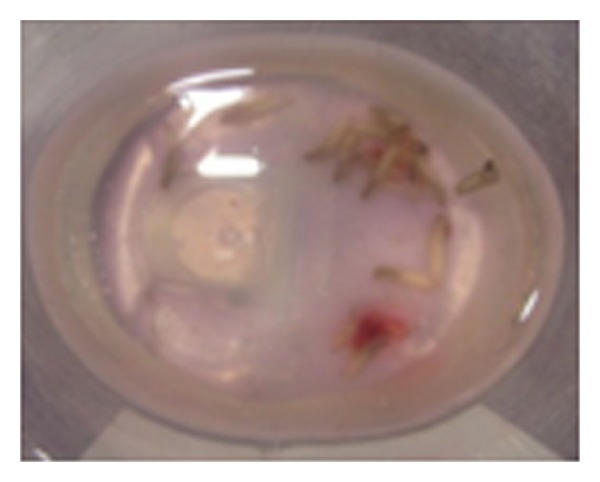
Larvae which was removed.

Cotton bud impregnated with turpentine oil was placed at the orifice of the socket for approximately 10 minutes. 14–16 maggots were manually removed with the help of hemostats (Figures 3 and 4). Patient administered Inj Amoxycillin + Potassium Clavulanate and Inj Metronidazole IV for 3 days. The larvae were mechanically removed for next three consecutive days with exploration, curettage, and warm saline irrigations till no further larvae could be found. No attempt was made to administer antiparasitic drug Ivermectin.

## 3. Discussion

 Myiasis of orodental complex is a rare entity commonly caused by common Indian housefly Musca Nebulo. They are found commonly in human habitats with poor hygiene and passable sanitation especially during summer and rainy season. Clinically, myiasis can be classified as primary or secondary [10]. They are based upon host dependence [11], mode of infestations, and anatomic sites [12]. Primary myiasis is caused by larvae that feed on living issue (biophagous). This form of myiasis is commoner in cattle and rare in humans. Secondary myiasis is caused by flies that feed on dead tissue (necrobiophagous). This is the more common type and infests patients with lesions that have necrotic cavities [13]. Severity myiasis depends upon location of infestation, lesions, and tissue inflammation. Many species of Dipterous flies among the genera Chrysomya and Cochliomyia have been reported to be the most important obligatory myiasis among human and/or domestic animals [14, 15].

 The lifecycle of a fly commences with egg stage followed by the larval stage, the pupal stage, and finally the adult fly. The requisites for egg laying and survival of the larvae are moisture, necrotic tissue, and suitable temperature. Thus wounds, open sores, scabs, and ulcers contaminated with discharges make possible way for the same. Modes of infestation in humans may occur in two ways, either accidentally with direct inoculation by the fly or by ingestion of infected material such as meat. In the present case, the location of the lesion is in the anterior part of the maxilla, implying a direct inoculation of the tissues.

 The condition is frequently associated with mental deficiencies [10, 16–18], where dexterity and impetus for maintaining oral hygiene are poor. Although the disease affects children commonly, a few cases of oral myiasis in children less than three years of age have also been reported [19]. These patients are completely dependent on their caregivers; however, in situations of low socioeconomic level these caregivers tend to neglect the oral hygiene of the patients because of the lack of health education [3]. The patient's precarious oral hygiene and halitosis are the probable risk factors in the present case in addition to its mental status.

 During the lifecycle of the parasite, the developmental transition via the larval stage, at all times, requires an intermediate host. The prevailing oral hygiene status provided the suitable substrate and temperature for the larvae. The stage of larvae lasts for six to eight days during which they are parasitic to human beings. They are photophobic and tend to hide deep into the tissues for a suitable niche to develop into pupa [20]. The present case also showed the larvae burrowed deep inside the wound in upper labial vestibule.

 Standard guidelines for management of oral myiasis do not exist, but more than a few authors note that the ideal approach is to remove all larvae and perform surgical debridement [11, 21]. The treatment incorporated in our case was simple and involved usage of antilarval measures (turpentine oil) followed by removal of the larvae [22]. 

 Treatment approaches other than procedural removal of the maggots include occlusion and administration of larvacides [11]. These techniques can be used in addition to manual removal, or if manual removal is not possible. By depriving larvae of oxygen, occlusion of an infested wound either kills the larvae or induces them to move more superficially where they can be removed more easily. Occlusion of infested wounds with a variety of substances has been described, including petroleum, nail polish, animal fat, beeswax, paraffin, hair gel, and mineral oil with varied success [11]. 

Early diagnosis, with adequate and careful surgical exploration of the lesion, seems to forestall extensive tissue damage and morbidity, and the necessity for complex surgical repairs that might be indicated at late stages of the disease. Early surgical treatment of oral myiasis is also important where esthetics is an issue, as in the present case. When there are multiple larvae in a single focus, and in advanced stages of parasitic development and tissue destruction, surgery should be complemented by treating the defect with turpentine oil or a comparable solvent capable of irritating the parasites and forcing them out of hiding. The possibility of oral myiasis should come to mind in relation to oral mucosal swellings with no apparent diagnosis in patients from areas where parasites that cause myiasis are endemic.

## 4. Conclusion

Oral myiasis is a rare and preventable disease. It can be prevented by controlling fly population, maintaining good oral and personal hygiene such as reducing the decomposition odor, cleaning and covering the wounds, and educating the susceptible population where basic sanitation is of low standard.

## References

[1] Hope FW (1840). On insects and their larvae occasionally found in human body. *Transactions of the Entomological Society of London*.

[2] Zumpt F, Zumpt F (1965). Myiasis in man and animals in the old world. *A Textbook For Physicians, Veterinarians and Zoologists*.

[3] Abdo EN, Sette-Dias AC, Comunian CR, Dutra CE, Aguiar EG (2006). Oral myiasis: a case report. *Medicina Oral, Patología Oral y Cirugía Bucal*.

[4] Aguiar AM, Enwonwu CO, Pires FR (2003). Noma (cancrum oris) associated with oral myiasis in an adult. *Oral Diseases*.

[5] Al-Ismaily M, Scully C (1995). Oral myiasis: report of two cases. *International Journal of Paediatric Dentistry*.

[6] Bozzo L, Lima IA, de Almeida OP, Scully C (1992). Oral myiasis caused by sarcophagidae in an extraction wound. *Oral Surgery Oral Medicine and Oral Pathology*.

[7] Lata J, Kapila BK, Aggarwal P (1996). Oral Myiasis. A case report. *International Journal of Oral and Maxillofacial Surgery*.

[8] Yoshitomi A, Sato A, Suda T, Chida K (1997). Nasopharyngeal myiasis during mechanical ventilation. *Japanese Journal of Thoracic Diseases*.

[9] Prabhu SR, Praetorius F, Sena Gupta SK, Prabhu SR, Wilson DF, Daftary DK, Johnson NW (1992). Myiasis. *Oral Diseases in TheTropics*.

[10] Shinohara EH, Martini MZ, de Oliveira Neto HG, Takahashi A (2004). Oral myiasis treated with ivermectin: case report. *Brazilian Dental Journal*.

[11] McGraw TA, Turiansky GW (2008). Cutaneous myiasis. *Journal of the American Academy of Dermatology*.

[12] Bhatt AP, Jayakrishnan A (2000). Oral myiasis: a case report. *International Journal of Paediatric Dentistry*.

[13] Ribeiro FAQ, Pereira CSB, Alves A, Marcon MA (2001). Tratamento da miíase humana cavitária com ivermectina oral. *Revista Brasileira de Otorrinolaringologia*.

[14] Srinivasan R, Pani SP (1992). Myiasis in human filarial lymphedema. *The Southeast Asian Journal of Tropical Medicine and Public Health*.

[15] Lee HL, Yong YK (1991). Human aural myiasis. *The Southeast Asian Journal of Tropical Medicine and Public Health*.

[16] Anil S, Jacob OA, Hari S (1989). Oral myiasis: a case report. *Annals of Dentistry*.

[17] Rossi-Schneider T, Cherubini K, Yurgel LS, Salum F, Figueiredo MA (2007). Oral myiasis: a case report. *Journal of oral science*.

[18] Carvalho RW, Santos TS, Antunes AA, Filho JR, Anjos ED, Catunda RB (2008). Oral and maxillofacial myiasis associated with epidermoid carcinoma: a case report. *Journal of Oral Science*.

[19] Droma EB, Wilamowski A, Schnur H, Yarom N, Scheuer E, Schwartz E (2007). Oral myiasis: a case report and literature review. *Oral Surgery, Oral Medicine, Oral Pathology, Oral Radiology, and Endodontics*.

[20] Roszalina R, Rosalan R (2002). Oral Myiasis: case report. *Malaysian Journal of Medical Sciences*.

[21] Antunes AA, de Santana Santos T, Avelar RL, Neto ECM, MacEdo Neres B, Laureano Filho JR (2011). Oral and maxillofacial myiasis: a case series and literature review. *Oral Surgery, Oral Medicine, Oral Pathology, Oral Radiology and Endodontology*.

[22] Singh I, Gathwala G, Yadav SP, Wig U (1991). Ocular myiasis. *Indian Pediatrics*.

